# Amorphous Titanium Dioxide Nanoparticles and Their Unexpected Fragmentation in MALDI-TOF/MS

**DOI:** 10.1021/acsomega.4c08770

**Published:** 2024-11-18

**Authors:** Artur L. Hennemann, Helton P. Nogueira, Miguel D. Ramos, Thiago C. Correra, Bruno L. Hennemann, Koiti Araki

**Affiliations:** Department of Fundamental Chemistry, Institute of Chemistry, University of São Paulo, 05508-000 São Paulo, SP, Brazil

## Abstract

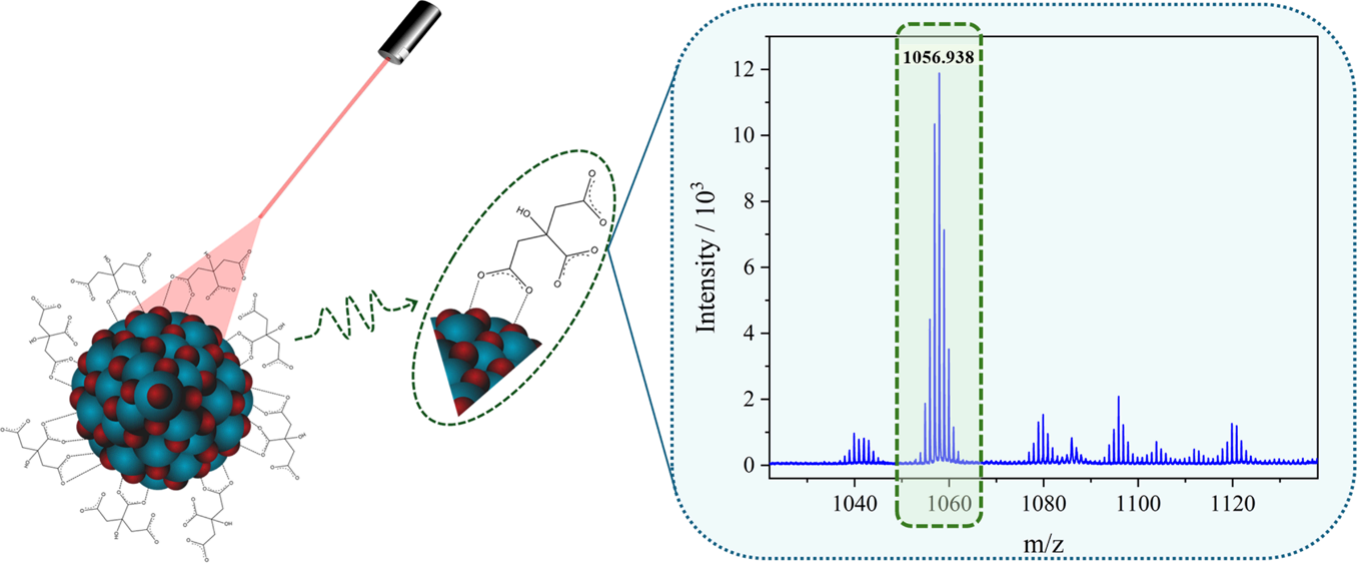

Amorphous 3 nm large ultrasmall (*us*TiO_2_) and 7 nm large anatase (*n*TiO_2_) nanoparticles (NPs) were successfully prepared and characterized by TEM, FTIR, DRX, UV–vis, and DLS techniques. The MALDI-TOF/MS was shown to be effective in assessing the surface chemistry but fragmentation processes precluded its use for evaluation of particle size distribution. In fact, the laser causes the fragmentation not only of amorphous TiO_2_ NPs but also of the material subjected to heat treatment and crystallization at 450 °C for 4 h upon interaction with the DHB matrix and TFA ionizing agent. No significant difference could be observed in the spectrum by varying the particle size, indicating the high stability of the TiO_2_ dimer and its low aggregates in the gaseous phase. In short, MALDI-TOF/MS is effective for the direct analysis of nanoparticle surfaces, especially the interaction of functionalizing molecular species with the inorganic components, which in combination with the other techniques demonstrated to be ideal for the detailed characterization of nanomaterials.

## Introduction

A revolution in nanotechnology has been witnessed over the past two decades driven by the exponential growth in the development and application of nanomaterials, more specifically, of engineered nanomaterials^1−4^ with controlled composition, size, and structure. In fact, new remarkable properties can be earned by nanostructuring itself as well as by the myriad of possibilities of combination of nanoparticles with other materials, thanks to their nanoscale size, thus bridging the gap between bulk materials and individual molecules. Accordingly, their properties tend to fool the principles of classical physics and pure quantum mechanics.^5−7^

The unique and exceptional physical and chemical properties of NPs are not solely determined by their chemical composition but also by their size, morphology, and surface chemistry, which can be modified by attaching ionic and molecular species onto the surface, allowing the easy customization of their physicochemical and biological properties.^8−11^ As a result, the possibilities of application of NPs are becoming increasingly wider spanning crucial industrial economic sectors such as chemical and pharmaceutical, health and biomedical, agribusiness, food and beverages, and information technology, among others.^2,8−15^

Titanium dioxide (TiO_2_) is one of the most largely explored materials since its industrial production in the early 20th century, especially as a white pigment in the manufacture of paints, sunscreens, ointments, and toothpaste among other applications.^16^ Nevertheless, since the discovery that it can photocatalytically split water under ultraviolet light irradiation by Fujishima and Honda^17^ in 1972, TiO_2_ has also been extensively explored in solar cells, photocatalytic degradation of pollutants and water purification, photoreduction of CO_2_ and photocatalytic organic synthesis.^18−21^ In this context, titanium dioxide-based nanomaterials have been eagerly pursued given their catalytic and photocatalytic activity enhanced by their expanded surface area.^22,23^ In addition, the reduction in size associated with morphological changes and nano structuration may lead to new physicochemical and optical properties, as well as fine-tuning of their catalytic properties.

Unveiling and understanding such unique properties generally require a multidisciplinary approach, utilizing a range of complementary techniques, thus paving the way for their utilization in diverse applications. Typically, microscopy techniques such as scanning electron microscopy (SEM), and transmission electron microscopy (TEM) enable the direct visualization of their core size and morphology, nanostructure, and surface topology. Complementarily, spectroscopic techniques such as X-ray diffraction (XRD), Fourier transform infrared spectroscopy (FTIR), and Raman spectroscopy provide insights into their crystal structures, chemical compositions, and molecular interactions. Surface analysis techniques such as atomic force microscopy (AFM) and X-ray photoelectron spectroscopy (XPS) can offer detailed information about surface properties and composition. Additionally, scattering techniques such as dynamic light scattering (DLS) and thermogravimetric analysis (TGA) aid in determining particle size distribution, stability, and thermal behavior of nanomaterials.^24,25^ Although the techniques mentioned above are fundamental, none is capable of accurately providing the actual mass of each of those nanoparticles. Accordingly, mass spectrometry (MS), which is widely used in molecular sciences, is now emerging as a powerful tool for the characterization of nanoparticles^26^ and ultrasmall nanoparticles (1–3 nm large).^27^ In the late 1980s, the development of electrospray ionization (ESI) and laser desorption techniques based on methods such as matrix-assisted laser desorption ionization (MALDI) extended the ionization limit to macromolecules, a breakthrough in mass spectrometry.^28^ In parallel, mass spectrometers became more sensitive and accurate and with increasingly higher resolving power. Thus, soft ionization sources (ESI and MALDI) proved to be extremely versatile in ionizing and transferring intact NPs to the gas phase.^29,30^

Mass spectrometry can provide valuable information in addition to nanoparticle mass^31−33^ such as the amounts of ligands bound onto the surface as well as the metal–ligand bond energy. For example, Yan et al.^34^ used an HPLC-MS/ultraviolet/chemiluminescent (HPLC-MS/UV/CLND) nitrogen detection system to quantify the density of two types of ligands bound on AuNPs chosen among neutral, positively charged, negatively charged, hydrophobic, or hydrophilic molecules. The ligands were displaced from the AuNPs surface by treatment with an I_2_/I^–^ solution followed by centrifugation to separate them from the NP cores. The identity of each ligand was confirmed by MS while their amounts were determined by an online CLND detector using standard calibration curves.

The formation of Ti_*x*_O_2*x*_ clusters during the preparation of about 1 nm large titanium oxide NPs was shown by Guan et al.^35^ using MALDI-TOF-MS, demonstrating the capability of this technique for nanoparticle analysis, especially of subnanometric size clusters, hardly accessible by TEM. Complementarily, Yan et al.^36^ focused on the direct identification of the ligands bound at the nanoparticle surface by mass spectrometry. Magnetite (Fe_3_O_4_) and FePt nanoparticles protected by a monolayer or mixed molecular monolayer were utilized for that purpose, enabling qualitative and quantitative analyses of the functionalizing thiol and dopamine ligands by laser desorption/ionization mass spectrometry (LDI-MS). The results were validated by liquid chromatography coupled with the mass spectrometry (HPLC-MS) technique.

On the other hand, Dass^37^ demonstrated the feasibility of analyzing both the inorganic and organic components in isolated gold NPs aggregates by the MALDI-TOF/MS technique. Thus, the molecular formula Au_68_(SCH_2_CH_2_Ph)_34_ was assigned to a 14 kDa nanocluster constituted by a 49-atom Marks decahedral core, comprising 19 inner core atoms and 30 outer atoms chelated with staple motifs, whose predicted formula is [Au]_19+30_ [Au(SR)_2_]_11_ [Au_2_(SR)_3_]_4_. This demonstrated the exceptional capability of MALDI-TOF/MS spectrometry in analyzing quite high mass nanostructures, opening new perspectives in nanoparticle characterization.

Yang et al.^38^ reported that, in addition to the physical shape information provided by fingerprint enhancement techniques (FET), recent studies have shifted focus toward the chemical composition of fingerprint residues. They utilized electrospray deposition to softly apply TiO_2_ NPs onto fingerprints without damaging their morphology. TiO_2_ NPs were also shown to be effective for matrix-assisted laser desorption ionization mass spectrometry imaging (MALDI MSI), enabling the detection of both endogenous and exogenous substances in fingerprints.

Accordingly, this technique was utilized to access the size and size distribution of citrate stabilized amorphous ultrasmall TiO_2_ (*us*TiO_2_) and 7 nm large nanoparticles (*n*TiO_2_), based on the mass-to-number of atoms relationship. Among mass spectrometry techniques, MALDI-TOF/MS is the most suitable for analyzing compounds containing titanium.^39,40^ Although previous studies have used MALDI-TOF to correlate peak maxima^40^ with nanoparticle size, our work demonstrates that this technique, while suitable for surface chemistry analysis, is challenging in directly correlating particle size with mass spectra due to significant fragmentation, even after thermal treatment and crystallization (*n*TiO_2_-450). In fact, the number and intensity of peaks below 2000 *m*/*z* 2000 were unexpectedly higher than expected by TEM and DLS data, revealing that laser-induced fragmentation can disrupt that correlation.

What distinguishes our work is the novel focus on these low *m*/*z* signals as indicators of critical surface chemistry characteristics such as the stability of Ti–O–Ti bonds and the interaction between ligands and the nanoparticle surface. Instead of pursuing particle size distribution through MALDI-TOF/MS, we shifted the focus to the understanding of surface-related phenomena governing nanoparticle behavior under laser irradiation, an underexplored area yet. This approach provides new insights into nanoparticle surface stability and ligand bonding/interactions, which are vital for nanomaterial properties.

**Figure 1:**
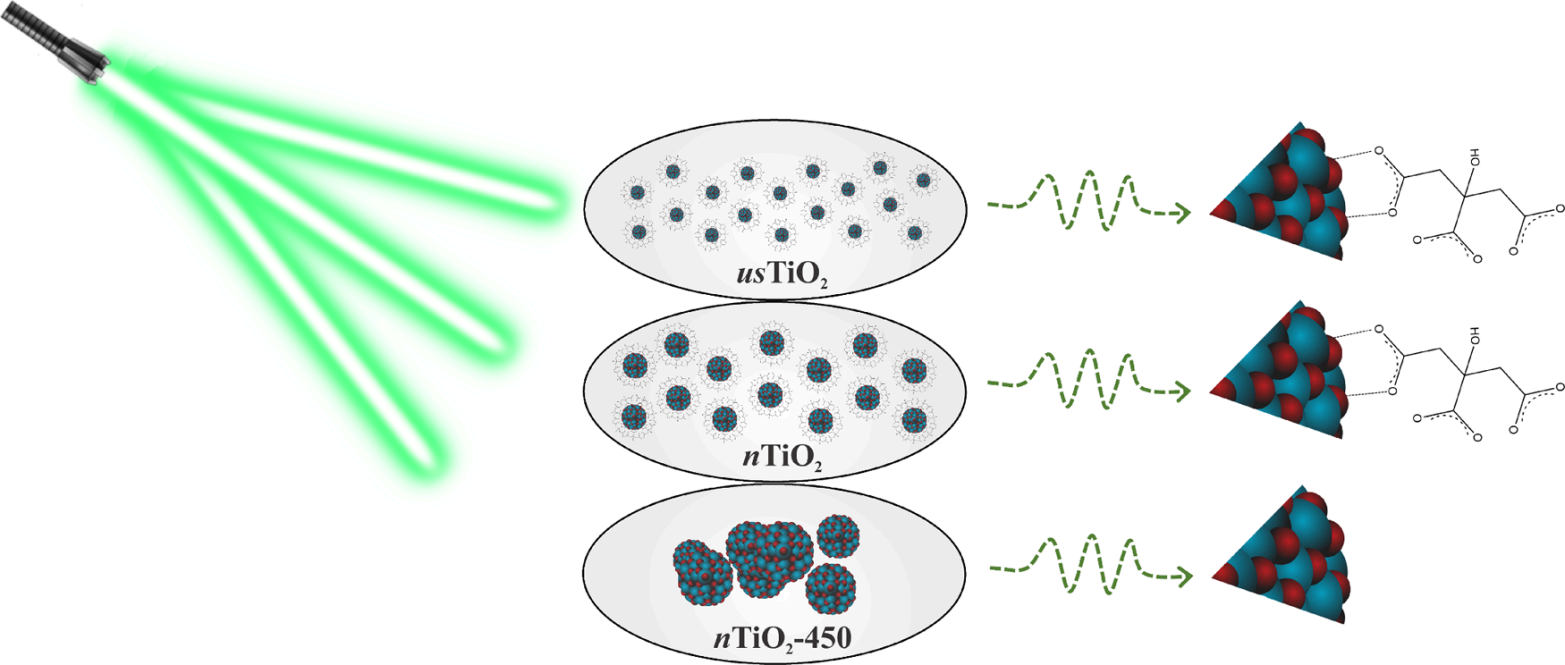
Illustration of MALDI-TOF/MS laser interaction with TiO_2_ NPs.

Our work demonstrates the potential of MALDI-TOF/MS not just for nanoparticle size analysis but as a powerful tool for probing surface chemistry, particularly in conjunction with complementary techniques such as TEM and DLS. The MALDI-TOF/MS laser interaction with TiO_2_ NPs is illustrated in Figure 1.

## Experimental Section

### Chemicals

Ultrapure Milli-Q DI-water was used throughout. Methanol (HPLC) and 2,5-dihydroxybenzoic acid (DHB) were purchased from Sigma-Aldrich and used without further purification. Titanium(IV) tetrachloride 99.9% (TiCl_4_) and citric acid were purchased from Sigma-Aldrich and Synth, respectively.

### Materials Synthesis

The nanoparticles were prepared according to Rab et al.^41^ by hydrolysis of TiCl_4_ in the presence of citric acid (Supporting Information, Figure S1) by varying the mixture rate and temperature to control size, and then the pH adjusted to 9–10 with NH_4_OH and refluxed to get *us*TiO_2_ and *n*TiO_2_ colloidally stabilized by citrate. They were precipitated out with ethanol and separated by centrifugation (4500 rpm for 15 min) to remove the contaminants in the solution. The solid was resuspended in DI-water, the previous precipitation/washing purification process with ethanol was repeated three more times, and the white solid product was dried under vacuum overnight.

### Physico-Chemical Characterization

The TiO_2_ nanoparticles were characterized by Fourier-transform infrared spectroscopy (FTIR), UV–vis spectroscopy, dynamic light scattering (DLS), X-ray diffractometry (XRD), and transmission electron microscopy (TEM).

The FTIR spectra were obtained in a Bruker ALPHA FTIR spectrophotometer in absorbance mode For the analysis, the sample solids were dispersed in dry KBr, and subsequently, pellets were prepared for measurement. The UV–vis spectra were registered in an Agilent HP8453A UV–vis diode-array spectrophotometer using a 10.0 mm optical path quartz cuvette, and a volume of 3.5 mL was employed for these measurements. The instrument covers a spectral range from 190 to 1100 nm with a resolution of 1 nm. The DLS and zeta potential analyses were carried out in a Zetasizer Nano S equipment (Malvern, UK) at 25°C, using a conventional quartz cuvette (10.0 mm) for the size measurements and specialized cuvettes equipped with gold electrodes for the zeta potential measurements. Typically, a volume of 1 mL was used for the size measurements and 0.9 mL for the zeta potential measurements. The XRD was obtained in a Bruker D8 Phaser diffractometer equipped with a Cu Kα source (λ = 1.5418 Å, 40 kV, 40 mA, step = 0.05°, setting the time step to 1 s) in the 2θ range from 5 to 90°. The TEM images were registered in a JEM 2100 JEOL transmission electron microscope, equipped with a LaB_6_ filament as an electron beam source. The polydispersity index (PDI) was calculated using data from TEM images according to eq 1,^42^ as recommended in ISO 9276–2:2014, where the standard deviation (σ) and the average diameter (D̅) of the particles are utilized.
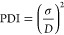
1

A matrix-assisted laser desorption/ionization (MALDI) time-of-flight (TOF) Ultraflextreme Bruker Daltonics mass spectrometer was used in positive and negative scan modes for mass spectra data acquisition in MS and MS/MS modes (reflector mode). The laser was set to 23% intensity in both positive and negative scan modes and to 27% in collision-induced dissociation (CID) mode, while the mass range was set from 50 to 5000 Da.

All of the samples were prepared by dispersing 2 mg of TiO_2_ nanoparticles in 1.0 mL of ultrapure DI-water and adding 1.00 μL of trifluoroacetic acid (TFA). Subsequently, an aliquot was diluted with a 5 mg/mL 2,5-dihydroxybenzoic acid (DHB) solution such that the final samples would be 1:10 w/w of TiO_2_ NPs to DHB, the best condition found upon careful optimization of the relative weight ratios and matrices. α-Cyano-4-hydroxicinnamic acid, dithranol, and sinapic acid matrices were also tested (Figures S2–S4), but DHB provided the best results and reported hereon. DHB was selected as the matrix for MALDI-TOF analysis of TiO_2_ nanoparticles due to its ability to coordinate onto the surface of the nanoparticles,^43^ enhancing ionization and enabling accurate detection.

## Results and Discussion

### Characterization of NPs

**Figure 2:**
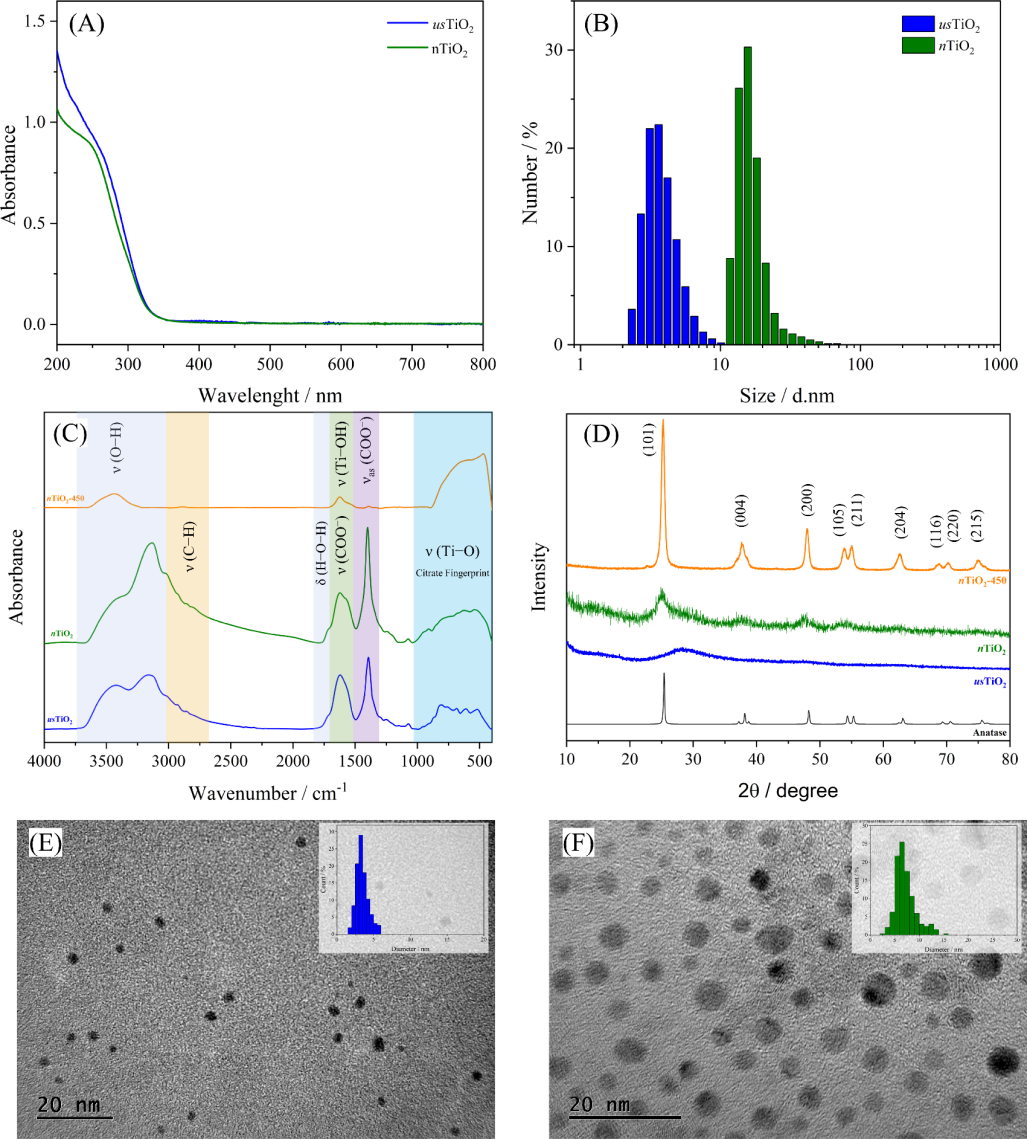
(A) UV–vis spectrum of the *us*TiO_2_ and *n*TiO_2_ and (B) size distribution histograms by number determined by DLS analysis of the *us*TiO_2_ and *n*TiO_2_ dispersions in water. (C) Comparison and assignment of the FTIR spectrum of *us*TiO_2_, *n*TiO_2_ and *n*TiO_2_-450. (D) X-ray diffractograms of the TiO_2_ NPs samples compared with those of the standard anatase. TEM images and size distributions of (E) *us*TiO_2_ and (F) *n*TiO_2_.

The UV–vis spectrum (Figure 2A) of the *us*TiO_2_ and *n*TiO_2_ nanoparticles shows a similar profile and an absorption onset at 340 nm, only about 10 nm blue-shifted as compared to the typical absorption onset of 350 nm of the most prevalent anatase and rutile polymorphs of titanium dioxide. Such a shift can be assigned to size effects suggesting that both TiO_2_ NPs still have a band structure but with a slightly wider band gap as compared to the bulky material.^44^

DLS analyses (Figure 2B) were carried out to confirm the dispersibility, colloidal stability, and average hydrodynamic particle size in aqueous suspension. The solid *us*TiO_2_ promptly disperses in water, generating a transparent and colorless suspension, with an average particle size by a number of 3.87 nm and a polydispersity index (PDI) of 0.192. The functionalization with citrate conferred a negative zeta potential of −40.1 mV (Figure S5A) and high colloidal stability, generating aqueous suspensions with no apparent change in viscosity. No additional peaks could be found even when the suspension was analyzed by intensity (Figure S5A), indicating that the sample is constituted by colloidally stable monodisperse nanoparticles.

The *n*TiO_2_ nanoparticles also disperse in water, displaying an average particle size by number of 16.8 nm, a PDI of 0.166, and a zeta potential of −30.8 mV (Figure S5B) consistent with the presence of citrate as a functionalizing layer. Nevertheless, the suspension exhibits a whitish hue that is corroborated by the DLS histogram by intensity (Figure S5B), which indicates the presence of larger nanoparticles in small quantities. Furthermore, a discrepancy is noted when these results are compared with the size distribution obtained from Transmission electron microscopy (TEM) analysis. This inconsistency is attributed to the higher concentration of the sample used in the DLS analysis compared to the suspension used for the preparation of the sample for TEM analysis. Therefore, it can be inferred that higher concentrations can lead to the formation of nanoparticle agglomerates in the suspension.

The FTIR spectrum profile of nanoparticles was also similar (Figure 2C) featuring the characteristic free citrate symmetric and antisymmetric COO^–^ stretching mode bands respectively around 1400 and 1600 cm^–1^, suggesting that it should be bound on the nanoparticles’ surface through one or two of its three carboxylate groups. The C–H stretching vibration was observed in the 2820 to 3000 cm^–1^ range, whereas the broad bands in the 3000 and 3700 cm^–1^ range can be assigned to the stretching mode of the citrate ligand hydroxyl groups,^45^ as well as to adsorbed water molecules,^46^ and Ti–OH bonds on the nanoparticles surface. Additionally, both, *us*TiO_2_ and *n*TiO_2_, exhibited several peaks in the spectrum attributed to citrate in the 1600 to 400 cm^–1^ range, in addition to those expected for the metal oxide core.^47^ In short, our results are consistent with nanoparticles functionalized with citrate ligands thus conferring high negative zeta potential and colloidal stability in aqueous suspension.

X-ray diffractometry (XRD) was utilized to evaluate the crystalline phase of the nanoparticles (Figure 2D). The *us*TiO_2_ exhibits no characteristic titanium dioxide diffraction peaks but only low intensity very broad features, as expected for an amorphous material.^48^ The *n*TiO_2_ sample, however, displayed a diffraction pattern of the anatase phase^49^ whose low intensity and broad features can be assigned to the presence of quite small crystallites.^50^ As expected, the *n*TiO_2_-450 sample, generated upon calcination of *n*TiO_2_ at 450 °C for 240 min, showed a typical anatase DRX pattern with intense peaks, indicating the occurrence of structural reorganization, probably associated with aggregation/densification processes, generating larger crystalline nanoparticles. Notably, *n*TiO_2_-450 consistently exhibited a weak band at 1640 cm^–1^ in the FTIR spectrum (Figure 2D), which was assigned to the asymmetric stretching vibrational mode of OH groups bonded to titanium (Ti–OH),^51^ even after 4 h of calcination at 450 °C, whereas the characteristic band associated with the Ti–O stretching mode is distinctly discernible in the region below 1000 cm^–1^ in the FTIR spectrum of *n*TiO_2_-450.

Transmission electron microscopy (TEM) images were carefully registered to investigate the morphology and core size of the *us*TiO_2_ and *n*TiO_2_ NPs. As shown in Figure 2E, the *us*TiO_2_ NPs were found to be essentially spherical, with an average diameter of 3 nm, smaller than the hydrodynamic size obtained by DLS analysis, as expected.^52^ A total of 168 particles were considered in the size histogram by TEM, resulting in an average size of 3.43 nm with a standard deviation of 0.87 nm and a PDI of 0.064, as expected for a narrower monomodal distribution. Interestingly enough, the spherical *n*TiO_2_ NPs (Figure 2F) were revealed to have an average size of only 7 nm by TEM, instead of 16.8 nm determined by DLS weighed by number. The size histogram obtained by TEM considering a total of 337 particles resulted in an average size of 7.17 nm with a standard deviation of 2.09 nm and PDI of 0.085. This data suggests that *n*TiO_2_ exhibits a broader distribution of particle sizes, including larger particles, which is consistent with the DLS measurements. However, significant discrepancies are noted for the results from TEM and DLS which can be attributed to the inherent characteristics of each technique. TEM provides a direct measurement of individual particle sizes in a dry state, allowing for the precise determination of the core size. In contrast, DLS measures the hydrodynamic diameter of particles in suspension, which can be influenced by particle agglomeration and the dynamic behavior of aggregates. At higher concentrations, ideal for DLS analysis, particles are more likely to move collectively, resulting in larger apparent sizes. Additionally, the interaction of particles in suspension and the presence of stabilizing agents can further contribute to variations in size measurements by the two techniques.^53^ Additionally, DLS is particularly sensitive to aggregates, as larger nanoparticles scatter more light than smaller ones.^52^ This sensitivity can lead to size discrepancies between the DLS and TEM measurements. For example, Coleman et al.^54^ found that if around 1% of the particles in a sample are significantly larger (e.g., two to three times the average size of the other 99%), DLS results can be significantly inflated compared to TEM. In their study, a reference silica sample measured 42 nm by DLS but only 25 nm by TEM. This demonstrates how a small fraction of larger particles can disproportionately affect DLS measurements, highlighting the importance of accounting for aggregation when analyzing nanoparticle sizes by this technique. Using citrate as one of the stabilizers for Ag nanoparticles, Choudhury et al*.*^55^ also observed a 13 nm difference between the results from those techniques. This discrepancy was attributed to the ligand’s ability to form hydrogen bonds, which strongly interact with the medium and increase the hydrodynamic diameter. Furthermore, a meticulous examination of the HR-TEM images of *us*TiO_2_ (Figure S6) indicated the presence of diffraction planes and thus of crystallites, in contrast with the DRX pattern of the solid with no peaks, as expected for an amorphous material. This apparent discrepancy can be attributed to some crystallization taking place due to the local heating induced by the incidence of the highly focused electron beam during the TEM analysis.^56^

### MALDI-TOF/MS Spectra of TiO_2_ Nanoparticles

MALDI-TOF/MS technique has been demonstrated to be a powerful tool to investigate the particle size distribution and composition, as shown previously.^57^ Since the measurements are based on the *m*/*z* ratio, we considered MALDI-TOF/MS to be a suitable technique for characterizing amorphous nanomaterials like our *us*TiO_2_ and *n*TiO_2_ nanoparticles. Instead of relying solely on the isotopic pattern of titanium, we examined the overall *m*/*z* peaks in the spectra. These peaks represent a complex overlap of isotopes from Ti, O, and C. The observed spectral profile aligns with literature reports, where the presence of additional titanium atoms contributes to the characteristic pattern^35^ we detected. In this context, the positive and negative mode spectral patterns of pure DHB were registered to find out the characteristic matrix spectral profiles in the analytical conditions (Figure 5), thus avoiding erroneous conclusions due to false positives or false negatives.

**Figure 3:**
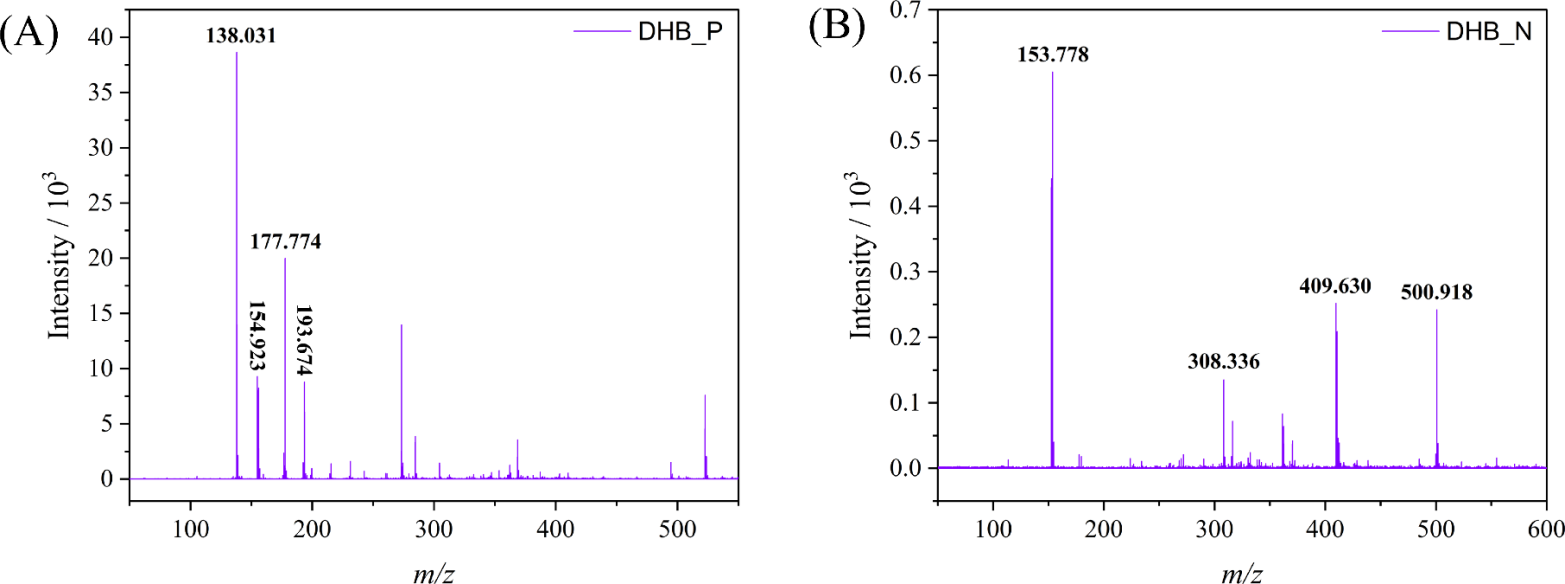
MALDI-TOF/MS spectrum of (A) positive and (B) negative mode DHB pattern with a laser at 23% intensity.

The peaks corresponding to the molecular ion (M + H^+^) at *m*/*z* 154.923, DHB + Na^+^ ion pair (*m*/*z* 177.774), DHB + K^+^ (*m*/*z* 193.674), and loss of a hydroxyl group from DHB (*m*/*z* 138.031) can be found in the MS spectrum of DHB in positive mode, as shown in Figure 3A. In the negative mode (Figure 3B), the peaks at *m*/*z* 153.778 and 308.336 corresponding to the DHB molecular ion (M – H)^−^, as reported by Gill et al.,^58^ and to the DHB dimer are the most significant ones. Monitoring those *m*/*z* species is fundamental to avoid considering false positives in the spectrum of the TiO_2_ nanoparticle samples of interest. The matrix DHB does not exhibit any signal in the mass spectrum above *m*/*z* = 600. Full mass spectra in both positive and negative modes, covering up to *m*/*z* 3000, are provided in the SI (Figures S7 and S8).

**Figure 4:**
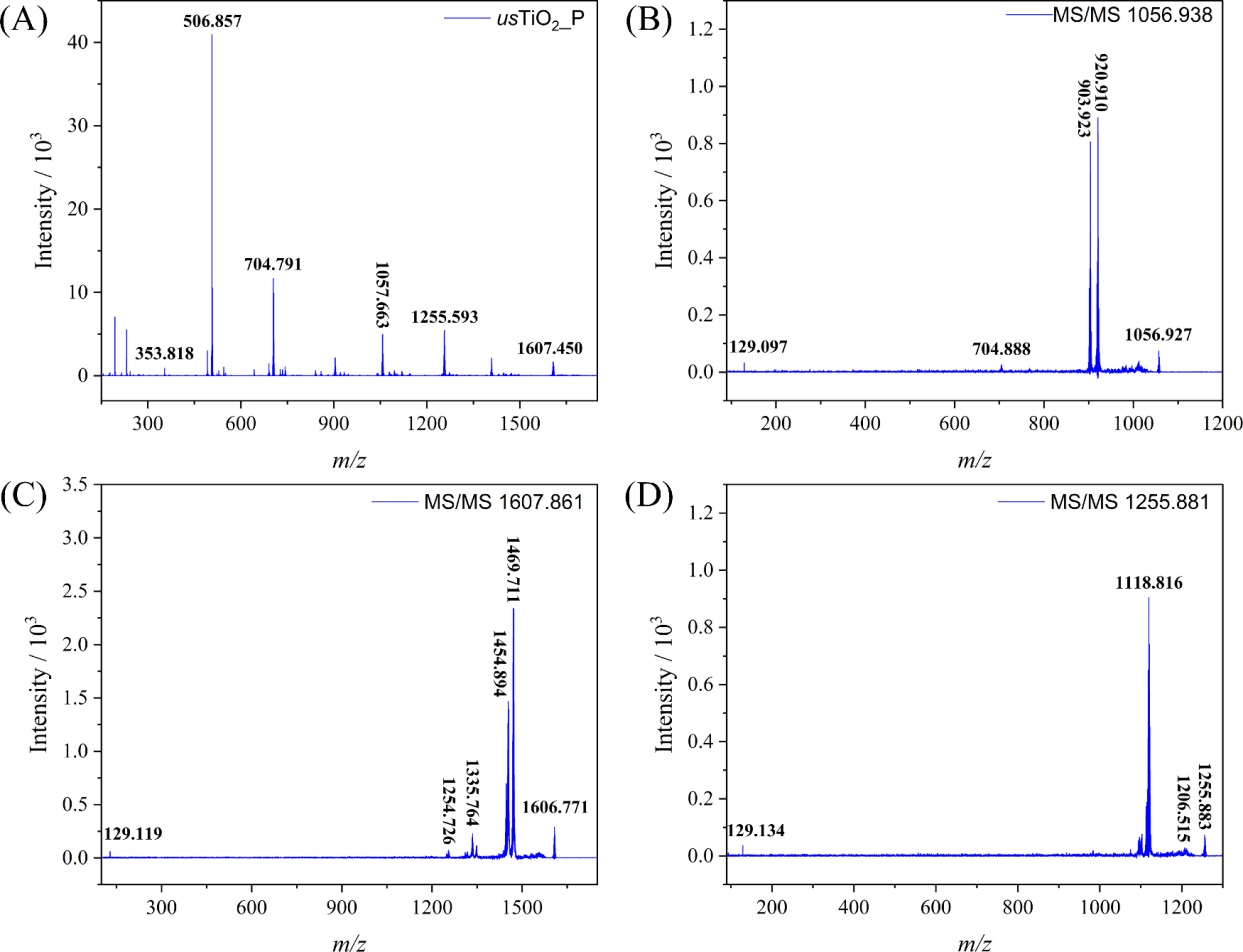
(A) Positive SCAN mode MALDI-TOF/MS spectrum of *us*TiO_2_ particles; and MALDI-TOF MS/MS positive spectrum of the (B*)m*/*z* 1056.938, (C) *m*/*z* 1607.861, and (D) *m*/*z* 1255.881 fragment.

The optimized spectrum of *us*TiO_2_ NP in positive SCAN mode depicted in Figure 4A exhibits peaks with *m*/*z* above 1000 Da, more specifically at *m*/*z* 1607.452, 1255.593, and 1057.663, that stand out from that of the DHB matrix. These fragments containing titanium atoms were selected and the product ions were generated in a subsequent MS analysis (MS/MS). The *m*/*z* 1056.938 species generated the *m*/*z* fragments at 920.910, 903.923, 704.888, and 129.097 (Figure 4B). The 920.910 ion corresponds to the loss of a 136 Da fragment^10^ by the precursor ion of *m*/*z* 1056.938 (1057 – 136 = 921), whereas the loss of a 154 Da fragment, corresponding to a DHB molecule, generates the *m*/*z* 903.92 ion. The fragment at *m*/*z* 136 is confirmed to originate from DHB, as MS/MS analysis of pure DHB in both positive and negative modes showed the generation of fragments at *m*/*z* 136 and 137. The MS/MS spectra of DHB are provided in SI (Figures S9 and S10). The 704.89 ion represents the loss of a fragment of 352 Da by the precursor ion (1057 – 352 = 705), in which the fragment with *m*/*z* 352 corresponds to a protonated (2TiO_2_ + citric acid) species. Finally, the *m*/*z* 191 fragment is typical of the citrate anion.^14^ Thus, the peak at 1056.927 nm corresponds to a protonated particle with composition 3(2TiO_2_ + citric acid).

The MS/MS spectrum of the cationic fragmentation with the highest *m*/*z* value of 1607.861 in the spectrum generated the fragments with *m*/*z* 1469.771, 1335.764, 1254.726, and, again, 129.119 (Figure 4C). The fragment with *m*/*z* 1469.771 corresponds to the loss of 129 Da, a fragment of DHB molecule, whereas the one at *m*/*z* 1254.726 and *m*/*z* 1335.764 corresponds to the loss of 352 and 272 Da, respectively, more specifically a protonated (2TiO_2_ + citric acid) and protonated (TiO_2_ + citric acid). Finally, the ion with *m*/*z* 1255.881 (Figure 4D) generated the fragments with *m*/*z* 1118.816 upon mass loss of 137 Da, corresponding to a fragment of DHB from the precursor ion.

At this moment, it is important to emphasize that all fragments of the molecular ion with *m*/*z* 1607.861 were constituted by 2TiO_2_ units bound to DHB and/or citrate ligand that appears as the *m*/*z* 129.134 peak in the spectra. The expected *m*/*z* values for larger TiO_2_ nanoparticles (3 and 7 nm) would typically be much higher due to the greater mass of the particles. For instance, theoretical *m*/*z* values for intact nanoparticles would be in the range of several thousand, depending on the number of TiO_2_ units. The observed *m*/*z* value of 1607.861 is lower than anticipated, suggesting that the detected ions likely result from fragmentation rather than intact larger nanoparticles. Regarding the choice of matrix, while DHB is commonly used for many applications, it may not be optimal for analyzing larger molecules or clusters. We have considered alternative matrices, such as sinapinic acid; however, preliminary tests indicated that it did not significantly improve the detection of larger *m*/*z* ions for TiO_2_ nanoparticles in our MALDI-TOF/MS setup. The omnipresence of the DHB matrix clearly indicates that it can strongly bind onto the TiO_2_ nanoparticle surface, probably displacing citrate molecules. Assuming these as facts, it can be inferred that the ion with *m*/*z* 1607.861 probably corresponds to a fragment of larger nanoparticles since that *m*/*z* value is well below the expected one for 3 and 7 nm large TiO_2_ nanoparticles. Accordingly, scans up to 35000 Da were performed but no peaks above *m*/*z* 1607 could be found, demonstrating that only fragments of the nanoparticles can be observed by MALDI-TOF/MS technique in positive mode, in contrast with the results presented by Guan et al.^35^ The main fragments found in the positive mode spectra of *us*TiO_2_ are listed in Table 1. The theoretical versus experimental mass data are detailed in the SI (Table S1).

**Table 1:** Precursor Ions and Main Fragments Found in the Positive Mode *us*TiO_2_ Spectra

precursor ions and main fragments generated from positive mode *us*TiO_2_ spectra	
precursor ion (*m*/*z*)	product ion (*m*/*z*)
1056.938	920.910, 903.923, 704.888, 129.097
1255.881	1206.515, 1118.816, 129.134
1607.861	1469.711, 1454.894, 1335.764, 1254.726, 129.119

**Figure 5:**
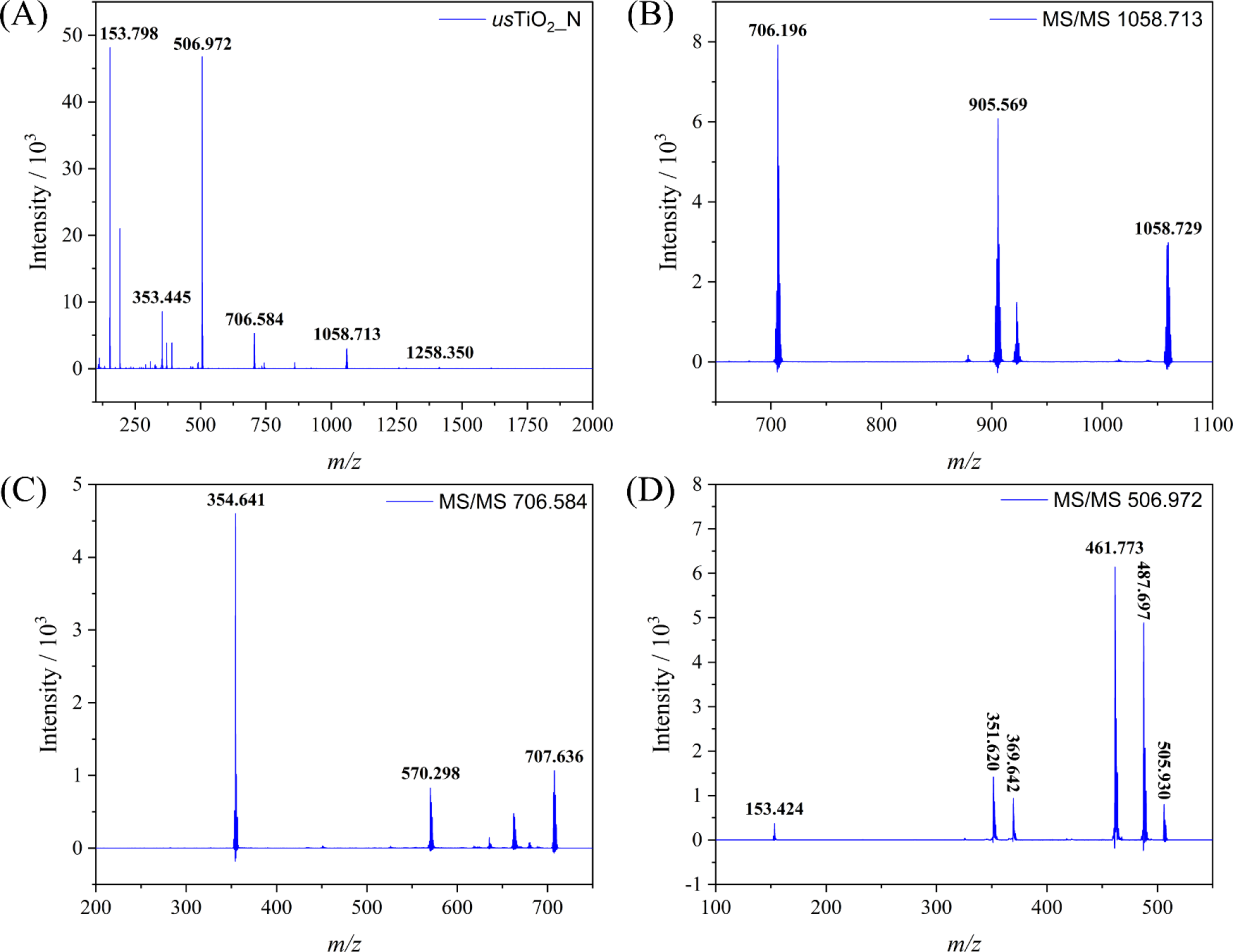
(A) Negative SCAN mode MALDI-TOF/MS spectrum of *us*TiO_2_ particles; MALDI/TOF MS/MS negative spectrum of the fragments with (B) *m*/*z* 1058.729; (C) *m*/*z* 707.636; and (D) *m*/*z* 505.930.

The negative mode MALDI-TOF/MS spectra were also registered to confirm the fragmentation process of TiO_2_ nanoparticles, just described above for the positive mode, generating (TiO_2_ + citrate) aggregates. The anion species with *m*/*z* 191.912 and 153.798 correspond to deprotonated citric acid and DHB, respectively, which could be detected at low laser powers of 23% suggesting the presence of bound citrate and DHB molecules on the surface. As expected, larger ions with *m*/*z* 706.584, 1058.729, and 1258.351 were also observed in the MALDI-TOF/MS spectrum of *us*TiO_2_ nanoparticles in negative SCAN mode, as shown in Figure 5A.

Analogously to the case of the spectrum obtained in the positive mode. The MS/MS spectra of the main fragments containing titanium were also obtained to understand their structure and composition. The *m*/*z* 1058.713 fragment ion generated product ions with *m*/*z* 922.579, 905.569, and 706.196 (Figure 5B). The *m*/*z* 922.579 ion implies the loss of a fragment of mass 136 by the precursor ion, corresponding to a DHB fragment. We further verified this by conducting MS/MS experiments on pure DHB, which demonstrated the generation of the fragment at *m*/*z* 136, confirming that this fragment indeed originates from DHB (Figures S9 and S10). The *m*/*z* 905.569 ion is generated by the loss of a deprotonated DHB molecule with a mass of 153, while the *m*/*z* 706.196 ion results from the loss of a mass of 352 au, corresponding to a protonated (TiO_2_ + citric acid) unit. Consequently, the precursor ion of *m*/*z* 1058.713 is composed of (6TiO_2_ + 3citrate) units, consistent with the results obtained in positive scan mode.

The product ion with *m*/*z* 706.584 was selected and analyzed generating product ions with *m*/*z* 570.298 and 354.641 (Figure 5C). The first one was assigned to the loss of a DHB fragment with mass 137 by the precursor ion, whereas the 354 ion was generated from the loss of a fragment of mass 353, i.e., (2TiO_2_ + citric acid). Similarly, the MS/MS spectrum of the product ion of *m*/*z* 506.972 (Figure 5D) generated the fragment ions with *m*/*z* 153.424 and 351.620 corresponding to deprotonated DHB and a fragment of (2TiO_2_ + citric acid), respectively. Interestingly, the *m*/*z* values of these fragments sum up exactly to *m*/*z* 506.972, which corresponds to a (2TiO_2_ + citric acid + DHB) unit. In short, the negative mode spectra showed a fragmentation pattern similar to that in positive mode spectra, and the main fragment peaks found in the negative mode spectra of *us*TiO_2_ are listed in Table 2.

**Table 2:** Precursor Ions and Main Fragments Found in the Negative Mode *us*TiO_2_ Spectra

precursor ions and main fragments generated from negative mode *us*TiO_2_ spectra	
precursor ion (*m*/*z*)	product ion (*m*/*z*)
1058.729	922.585, 905.569, 706.196
706.584	662.580, 573.371, 570.298, 354.641
506.972	490.715, 487.697, 464.745, 461.773, 369.642, 351.620, 153.424

Nanoparticles of varying sizes exhibited analogous mass spectra (*us*TiO_2_ and *n*TiO_2_) highlighting the capability of MALDI-TOF/MS to generate surface spectra of the nanoparticles. The mass spectra of *n*TiO_2_ are provided in SI, Figures S11 and S12.

To ensure the MALDI-TOF/MS system was operating properly for analysis of larger molecular mass ions, we performed MALDI-TOF/MS on cytochrome C, a protein with a molecular weight of 12 kDa (Figure S13). The successful detection of cytochrome C confirmed that the MALDI-TOF/MS system is capable of accurately measuring larger molecules. This observation indicates that the absence of higher mass peaks in our NP spectra is not attributable to the limitations of the MALDI-TOF/MS technique employed. Instead, the absence of higher *m*/*z* value species in our nanoparticle spectra is likely attributed to factors such as fragmentation processes rather than any inherent limitation of the MALDI-TOF/MS method itself.

**Figure 6:**
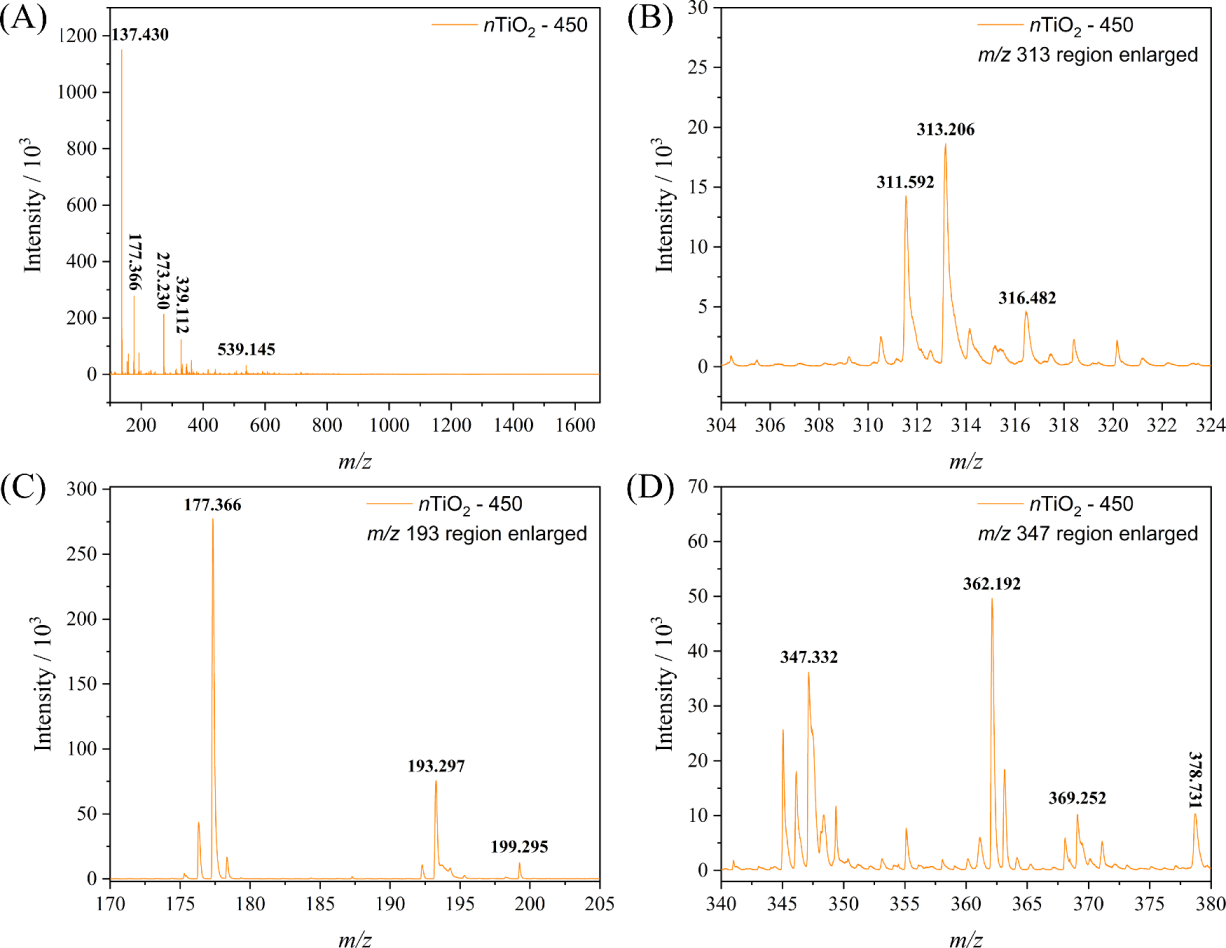
(A) Positive SCAN mode MALDI-TOF/MS spectrum of *n*TiO_2_-450 particles; and spectrum expanded at the (B) *m*/*z* 313.206, (C) *m*/*z* 193.297, and (D) *m*/*z* 347.332 range.

At this point, the fragmentation of our TiO_2_ nanoparticles probably is attributed to their amorphous nature and the possibility of forming aggregates of (2TiO_2_ + citric acid) units instead of crystallites with strong Ti–O bonds. This hypothesis was tested by subjecting *n*TiO_2_ nanoparticles to heat treatment at 450 °C for 4 h, open into air (*n*TiO_2_-450), thus completely eliminating the citrate ligand and adsorbed water, as confirmed by FTIR (Figure 2). In addition, the heating was enough to promote structural reorganization, inducing the formation of anatase nanocrystals and thus minimizing the presence of dangling TiO_2_ groups. Consequently, the peaks at *m*/*z* 1056.927 and 1607.451 in the positive scan mode spectrum of *us*TiO_2_ nanoparticles, constituted by TiO_2_ and citrate ligand, could not be found in the spectrum of *n*TiO_2_-450 (Figure 6A), and no peak with *m*/*z* > 539.145 was found. However, the peak at *m*/*z* 313.206 observed in Figure 6B, can be assigned to a fragment of (2TiO_2_ + DHB) indicating that the presence of strong coordinating DHB can promote the break of Ti–O bonds leading to the fragmentation of TiO_2_ nanoparticles during MALDI-TOF experiments. In addition, the peak at *m*/*z* 193.297 in Figure 6C demonstrates the possibility of the formation of (TiO_2_ + TFA) or (DHB + K^+^) fragments. Finally, the peak at *m*/*z* 347.332 in the *n*TiO_2_-450 spectrum, assigned to a protonated (TiO_2_ + DHB + TFA) fragment (Figure 6D), demonstrates that interactions with the matrix and the ionization agent can induce the fragmentation of TiO_2_ NPs and, probably, other oxide nanomaterials, making it difficult using the MALDI-TOF/MS technique for nanoparticle size analysis.

MALDI-TOF/MS presents distinct advantages compared to other techniques such as FT-IR and UV–vis, particularly in its capacity to detect surface-bound species with high sensitivity, even at low concentrations. Unlike UV–vis or DRS, MALDI is capable of differentiating molecules with similar chemical functionalities based on their mass, which proves to be especially useful in identifying surface impurities or chemically similar species that may exhibit overlapping vibrational features in FT-IR. Furthermore, MALDI offers the ability to directly analyze the surface chemistry of nanoparticles without the need for complex sample preparation, making it a highly complementary technique in the detailed characterization of nanomaterials.

## Conclusions

The TEM, DLS, DRX, UV–vis, and qualitative Tyndall effect analyses confirmed the successful preparation of amorphous 3 and 7 nm large *us*TiO_2_ and *n*TiO_2_ nanoparticles, whereas the FTIR and mass spectrometry results clearly showed the presence of functionalizing citrate ligand on the surface. A more in-depth study by the MALDI-TOF/MS technique indicated that the laser can cause the fragmentation not only of amorphous TiO_2_ NPs but also of materials subjected to heat treatment and crystallization upon interaction with functionalizing ligands, the DHB matrix, and the TFA ionizing agent, demonstrating that it should be used with caution. Nevertheless, the efficacy of that technique for direct analysis of nanoparticles surface, especially the interaction of molecular species with the inorganic component is highlighted. No significant difference in the spectrum could be observed when the particle size was increased, indicating the high stability of the TiO_2_ dimer and its low aggregates in the gaseous phase, providing significant advancement in the characterization of TiO_2_ nanoparticles by MALDI-TOF/MS.

MALDI-TOF/MS demonstrates significant advantages in the surface characterization of TiO_2_ NPs, particularly due to its high sensitivity and ability to detect low concentrations of surface-bound molecules. This technique allows for the differentiation of molecules with similar chemical structures, which FT-IR might struggle to distinguish, by leveraging the mass-specific nature of MALDI. Furthermore, MALDI-TOF/MS is capable of identifying impurities that may present overlapping vibrational bands in FT-IR, providing a more comprehensive analysis of surface chemistry. Thus, MALDI-TOF/MS serves as a valuable complementary tool for nanoparticle characterization, particularly when combined with other analytical methods.

In short, amorphous ultrasmall TiO_2_ nanoparticles were prepared and the MALDI-TOF/MS was shown to be a special technique among mass spectrometry techniques for their analysis. The technique was effective in assessing the surface chemistry of nanoparticles, but fragmentation processes precluded its use for the evaluation of nanoparticle size distribution. In fact, only low *m*/*z* fragments were detected validating our hypothesis of laser-induced fragmentation of TiO_2_ nanoparticles, even after thermal treatment and crystallization at 450 °C for 4 h.

## Dedication

This work is dedicated to the 75th birthday of Professor Henrique Eisi Toma, who devoted his life to pioneering research and mentorship at Institute of Chemistry of University of São Paulo.

## Abbreviations

DHB: 2,5-dihydroxybenzoic acid
DLS: dynamic light scattering
FTIR: Fourier-transform infrared spectroscopy
MALDI-TOF: matrix-assisted laser desorption ionization equipped with a time-of-flight
*n*TiO_2_: titanium dioxide 7 nm large nanoparticles
*n*TiO_2_-450: titanium dioxide 7 nm large nanoparticles after calcination at 450 °C for 4 h
TEM: transmission electron microscopy
TFA: trifluoroacetic acid
TiO_2_: titanium dioxide
*us*TiO_2_: titanium dioxide amorphous ultrasmall nanoparticle with 3 nm of size
XRD: X-ray diffractometry
