# Supplementary material for: Amorphous Titanium Dioxide Nanoparticles and Their Unexpected Fragmentation in MALDI-TOF/MS
Source: ACS Omega. 2024 Nov 18;9(48):47831–41. doi: 10.1021/acsomega.4c08770 (PMC11618435; doi:10.1021/acsomega.4c08770)
Supplement: Supplementary file 1 — ao4c08770_si_001.pdf [file ao4c08770_si_001.pdf]

-Supporting Information-

Amorphous Titanium Dioxide Nanoparticles and Their Unexpected Fragmentation in MALDI-TOF/MS

Artur L. Hennemann[a], Helton P. Nogueira[a], Miguel D. Ramos Jr.[a], Thiago C. Correra[a], Bruno L. Hennemann[a*], Koiti Araki[a*],

Department of Fundamental Chemistry, Institute of Chemistry, University of São Paulo. 05508-000 São Paulo, SP, Brazil.

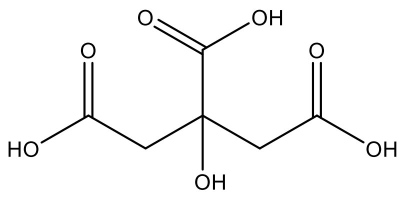

2-hydroxypropane-1,2,3-tricarboxylic acid
Chemical Formula: $C_6H_8O_7$
Molecular Weight: 192,12

Figure S1 – Structure and molecular formula of NPs stabilizing ligand.

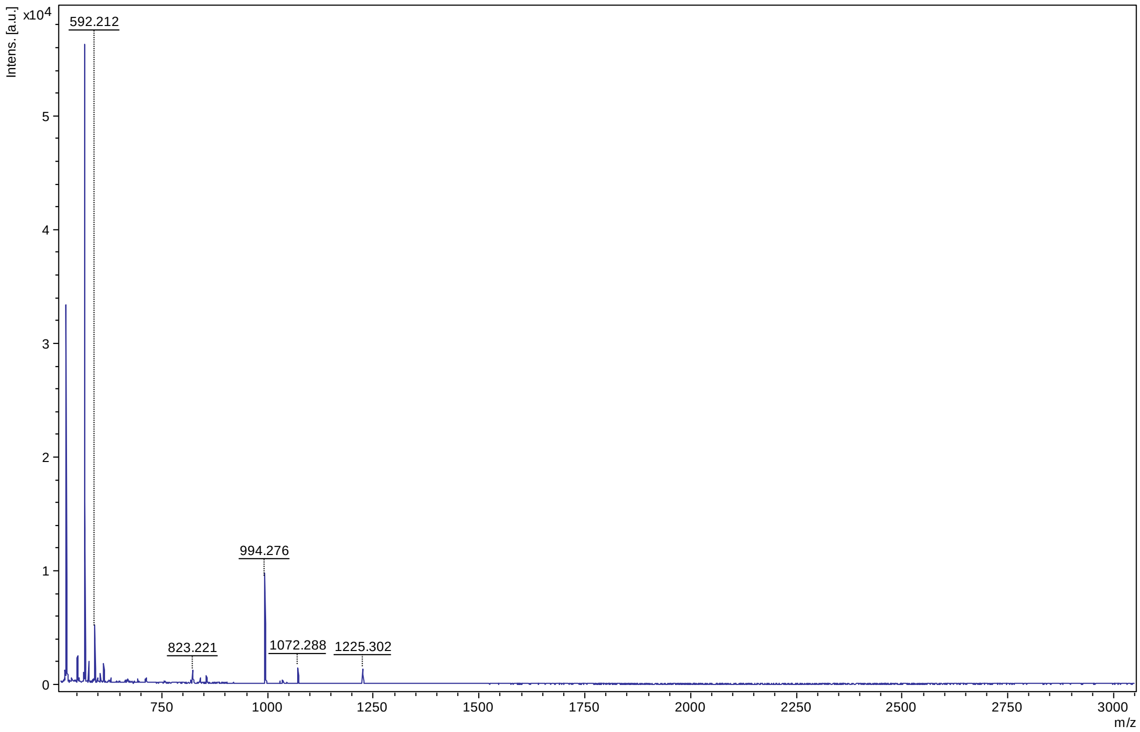

Figure S2 – Mass spectra of α-Cyano-4-hydroxycinnamic acid in positive scan mode.

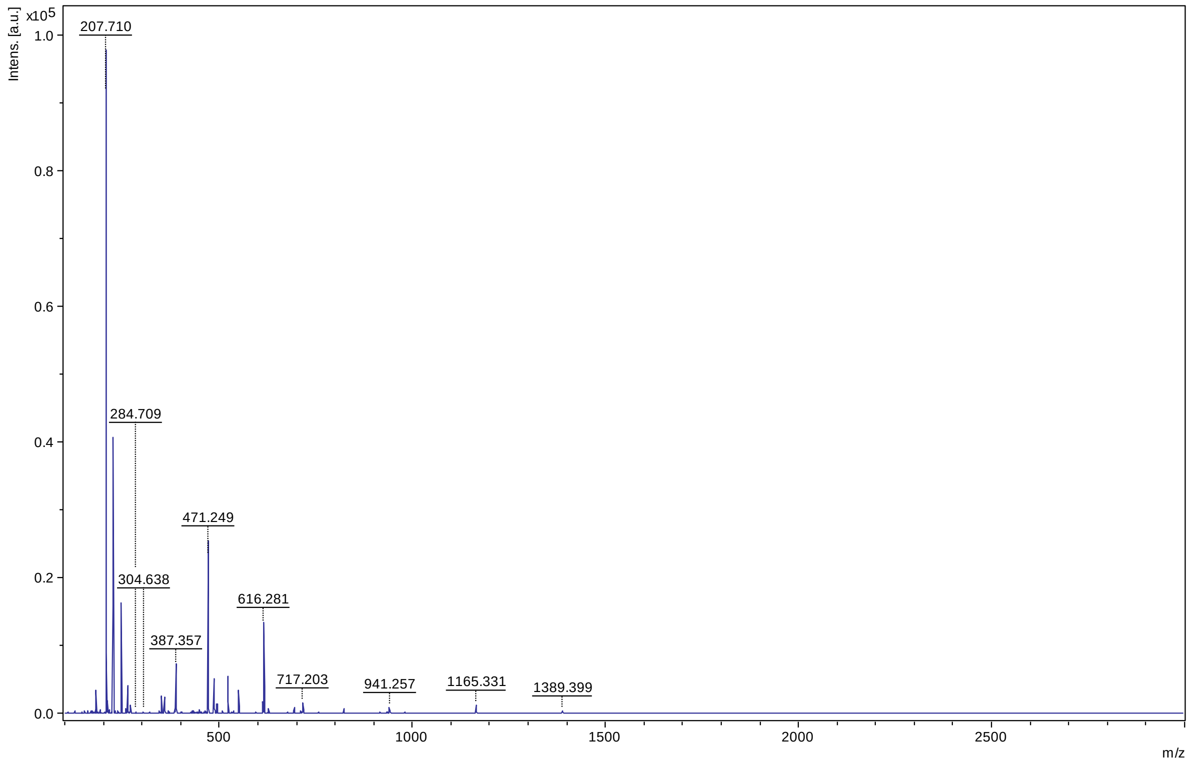

Figure S3 – Mass spectra of sinapic acid in positive scan mode.

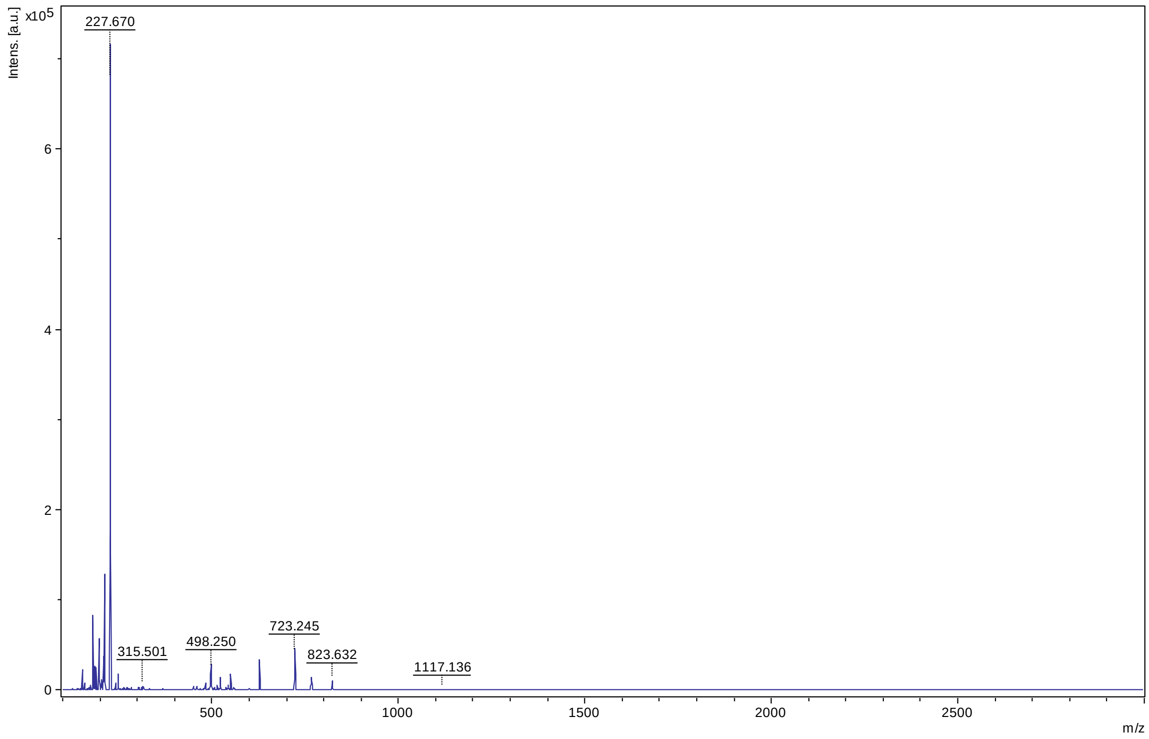

Figure S4 – Mass spectra of dithranol in positive scan mode.

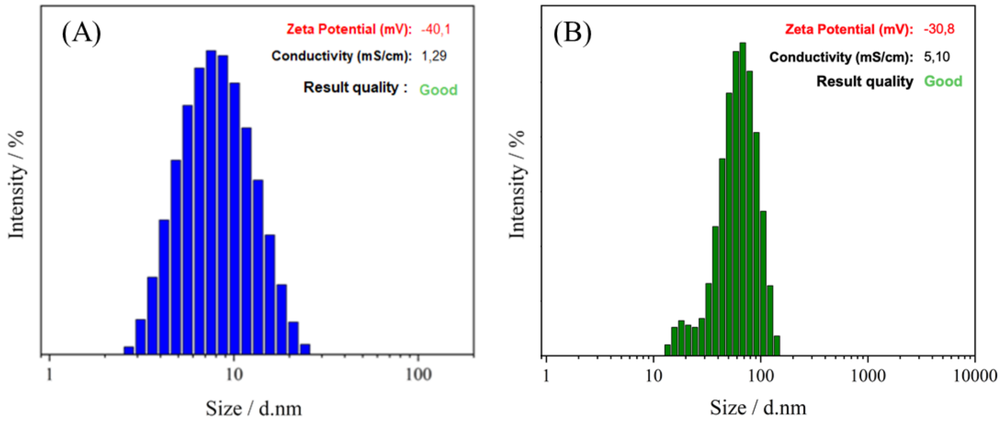

Figure S5 – DLS of (A) *us*TiO_2 (B) *n*TiO_2 weighed by intensity, along with their respective reports from the Zetasizer Nano S (Malvern, UK) for the Zeta potential measurements.

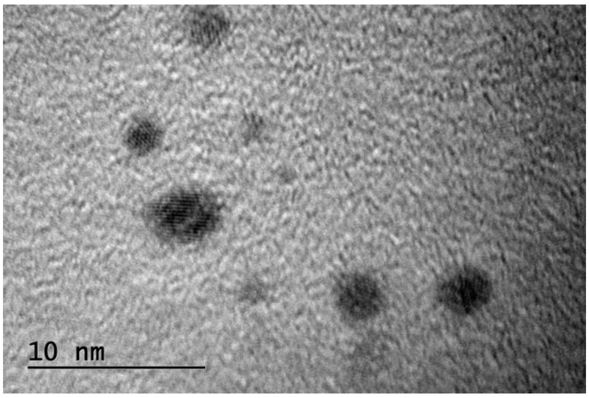

Figure S6 – HR-TEM image of *us*TiO_2.

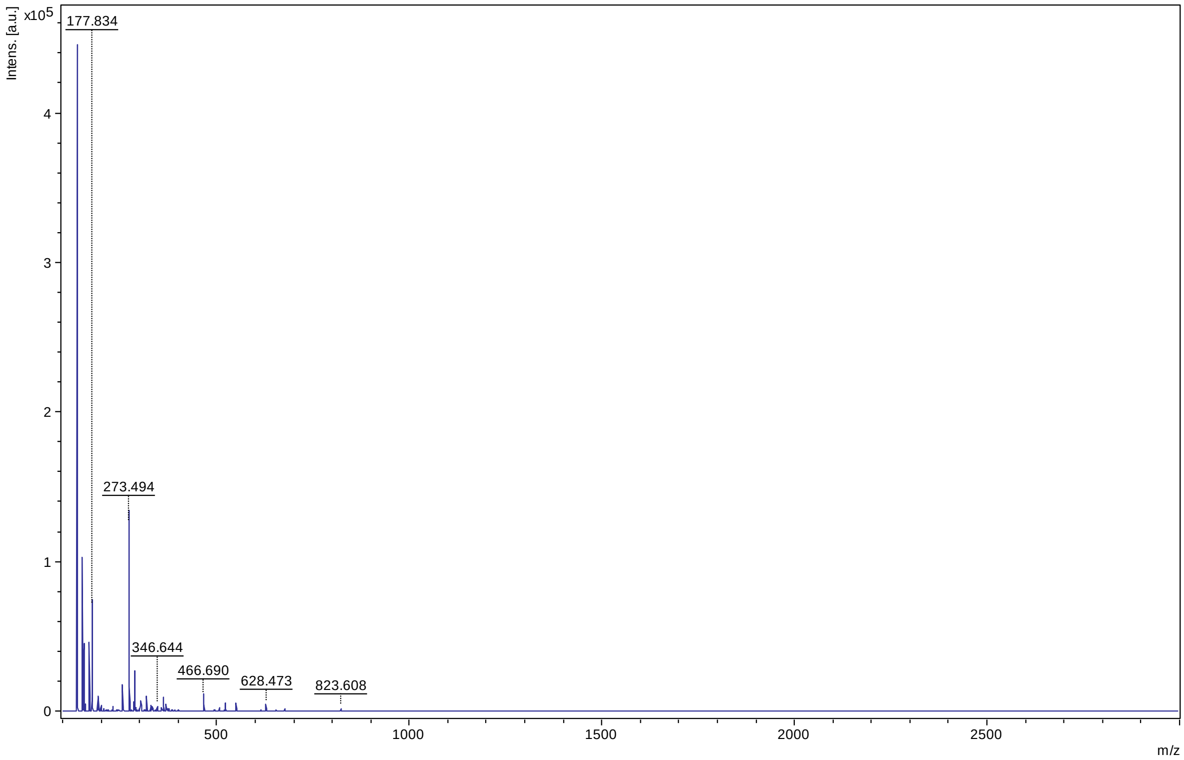

Figure S7 – Mass spectra of DHB in positive scan mode.

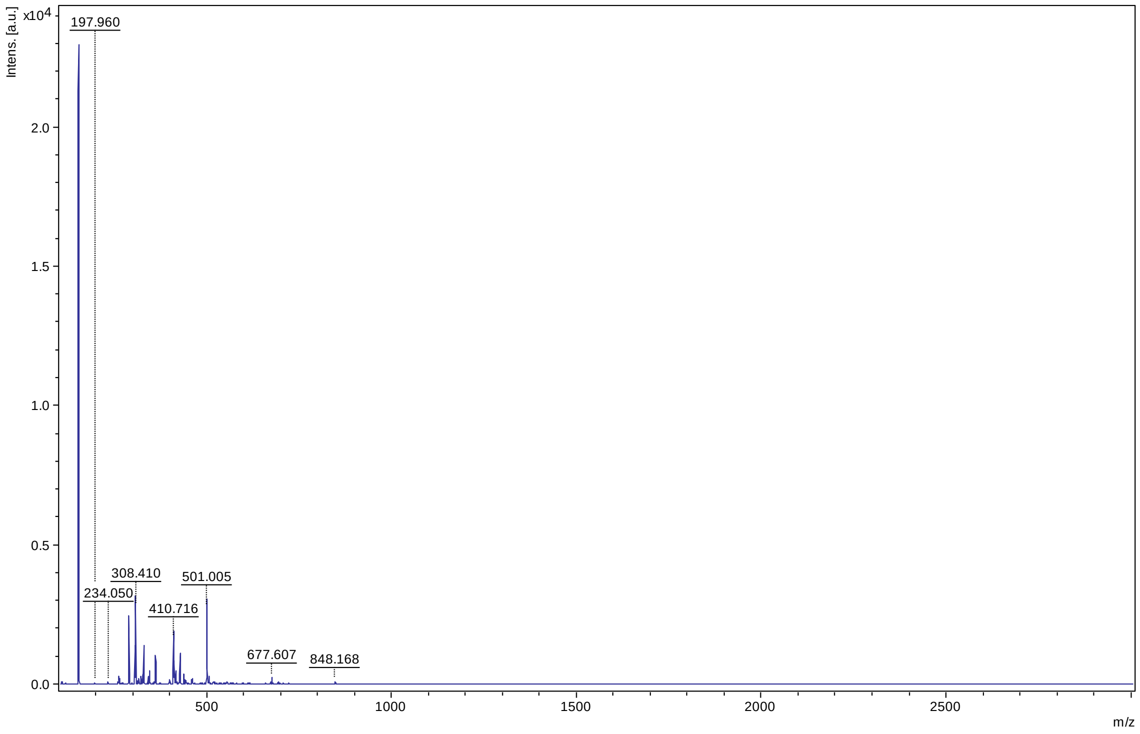

Figure S8 – Mass spectra of DHB in negative scan mode.

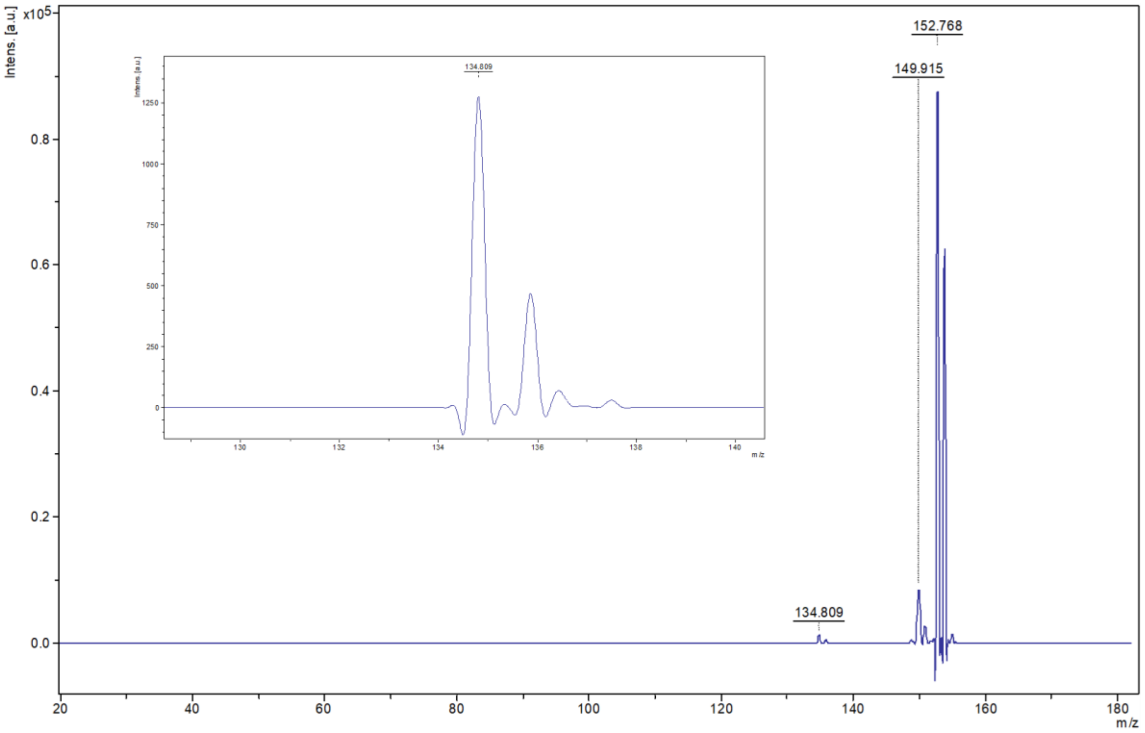

Figure S9 – Mass spectra of DHB in positive MS/MS mode.

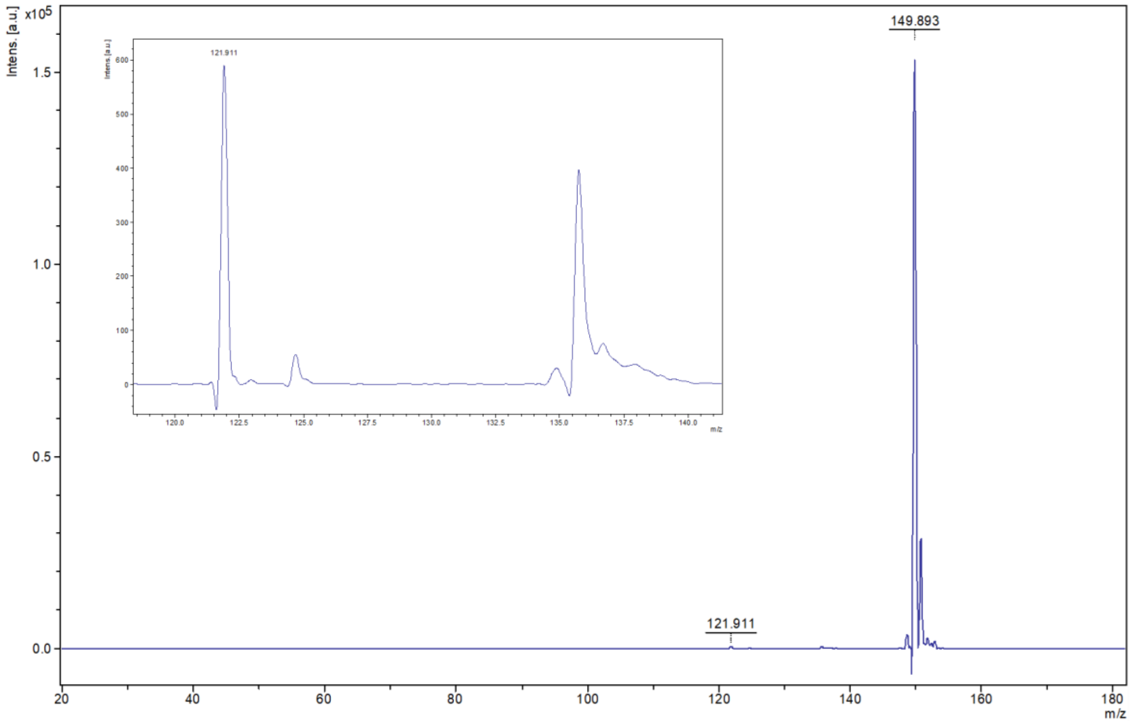

Figure S10 – Mass spectra of DHB in negative MS/MS mode.

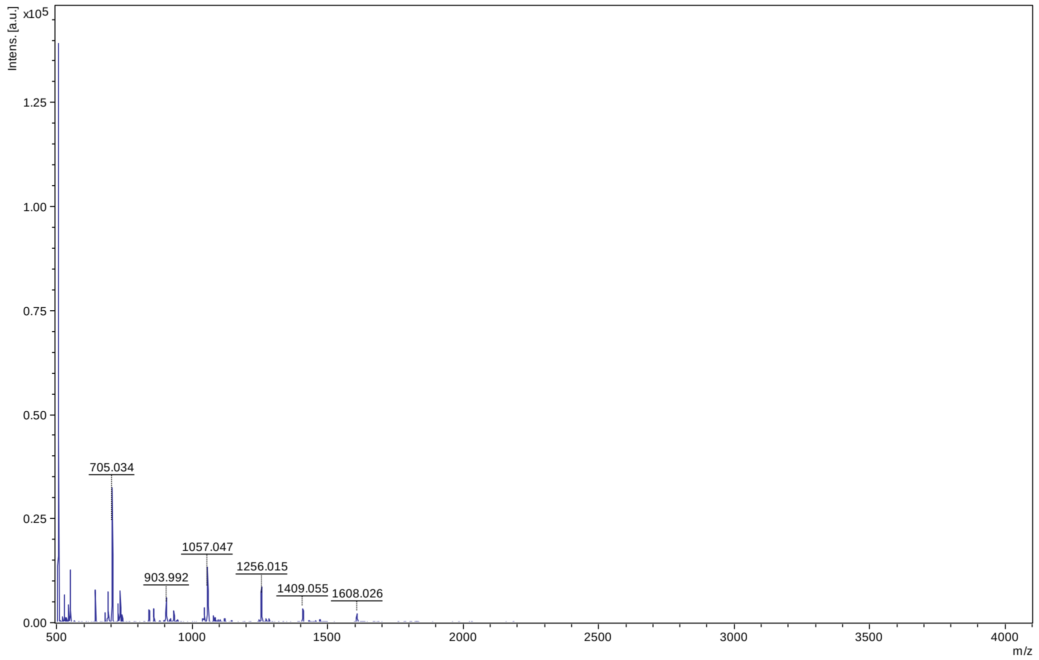

Figure S511 –Mass spectra of $n\mathrm{TiO_2}$ in positive scan mode.

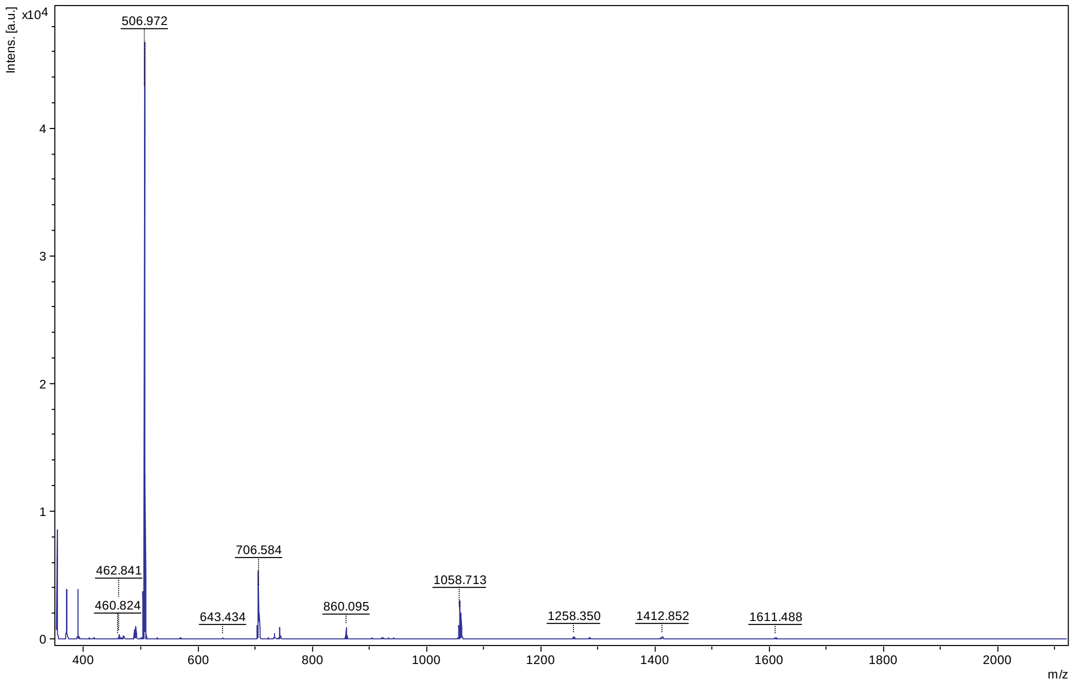

Figure S12 – Mass spectra of $n\mathrm{TiO_2}$ in negative scan mode.

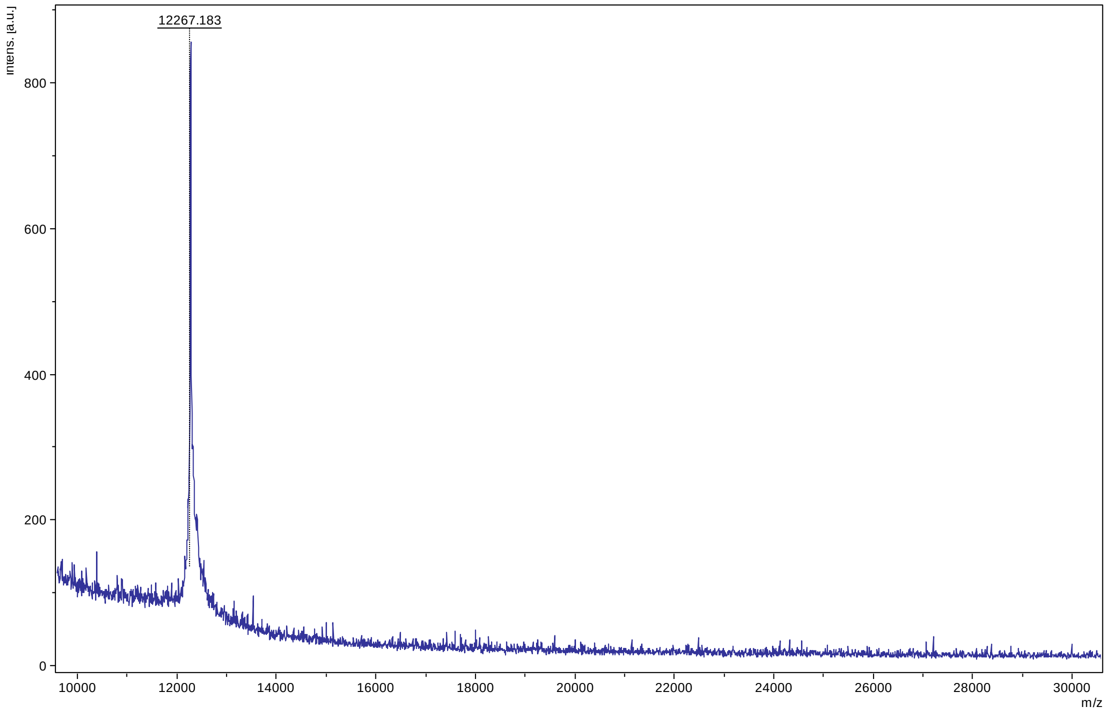

Figure S13 – Mass spectra of cytochrome-c in positive scan mode.

Table S1 – Actual versus Theoretical mass of ions.

Species	Actual mass (Da)	Theoretical mass (Da)	Deviation (Da)	Deviation (%)
DHB + Na^+	177.774	177.111	0.663	0.374
DHB + K^+	193.674	193.219	0.455	0.235
DHB + H^+	154.923	155.129	0.206	0.132
3(2TiO_2 + Citric Acid) + H^+	1056.988	1056.567	0.421	0.039
2(2TiO_2 + Citric Acid) + H^+	704.791	704.714	0.077	0.0109
2TiO_2 + Citric acid + DHB + H^+	506.857	506.982	0.125	0.024
2TiO_2 + Citric Acid + H^+	353.818	352.861	0.957	0.271
2TiO_2 + DHB + H^+	313.206	313.851	0.645	0.205
TiO_2 + DHB + TFA + H^+	347.332	347.008	0.324	0.093
DHB - H^+	153.778	153.113	0.665	0.434
